# Flexible parental care: Uniparental incubation in biparentally incubating shorebirds

**DOI:** 10.1038/s41598-017-13005-y

**Published:** 2017-10-16

**Authors:** Martin Bulla, Hanna Prüter, Hana Vitnerová, Wim Tijsen, Martin Sládeček, José A. Alves, Olivier Gilg, Bart Kempenaers

**Affiliations:** 1 Department of Behavioural Ecology & Evolutionary Genetics, Max Planck Institute for Ornithology, Seewiesen, Germany; 2 Department of Wildlife Diseases, Leibniz Institute for Zoo- and Wildlife Research, Berlin, Germany; 3 Department of Zoology, Faculty of Environmental Sciences, Charles University in Prague, Prague, Czech Republic; 4 Poelweg 12, 1778 KB Westerland, The Netherlands; 5 Department of Ecology, Faculty of Environmental Sciences, Czech University of Life Sciences, Prague, Czech Republic; 6 Department of Biology & Centre for Environmental and Marine Studies, University of Aveiro, Aveiro, Portugal; 7 South Iceland Research Centre, University of Iceland, Selfoss, Iceland; 8 Equipe Ecologie Evolution, UMR 6282 Biogéosciences, Université de Bourgogne Franche Comté, 6 Bd Gabriel, Dijon, 21000 France; 9 Groupe de Recherche en Ecologie Arctique, 16 Rue de Vernot, Francheville, 21440 France

## Abstract

The relative investment of females and males into parental care might depend on the population’s adult sex-ratio. For example, all else being equal, males should be the more caring sex if the sex-ratio is male biased. Whether such outcomes are evolutionary fixed (i.e. related to the species’ typical sex-ratio) or whether they arise through flexible responses of individuals to the current population sex-ratio remains unclear. Nevertheless, a flexible response might be limited by the evolutionary history of the species, because one sex may have lost the ability to care or because a single parent cannot successfully raise the brood. Here, we demonstrate that after the disappearance of one parent, individuals from 8 out of 15 biparentally incubating shorebird species were able to incubate uniparentally for 1–19 days (median = 3, *N* = 69). Moreover, their daily incubation rhythm often resembled that of obligatory uniparental shorebird species. Although it has been suggested that in some biparental shorebirds females desert their brood after hatching, we found both sexes incubating uniparentally. Strikingly, in 27% of uniparentally incubated clutches - from 5 species - we documented successful hatching. Our data thus reveal the potential for a flexible switch from biparental to uniparental care.

## Introduction

Parental care is a tremendously diverse social trait. The extent of parental cooperation varies along a continuum, from parents equally sharing all care to uniparental care in which either the female or the male provides all care^1,2^. Recent theoretical work and comparative empirical studies suggest that the sex that is in short supply in the population has increased mating opportunities, and is thus less likely to provide care than the more abundant sex^3–8^. Although empirical studies provide some support for the role of the adult population sex-ratio in shaping parental care patterns on an evolutionary time-scale, it is less clear whether individuals can flexibly adjust their patterns of parental care in relation to the environment, including the current population sex-ratio. Essentially, the species’ evolutionary history might have fixed the pattern of parental care, leaving little room for flexibility in who cares.

In some species, the caring sex varies between pairs (e.g. ref.^9–15^). For example in some cichlid fish, males are more likely to desert their brood when opportunities to breed are high^9,10^. In several bird species, biparental care is facultative (e.g. ref.^13–18^), whereas in others it is considered obligatory^19,20^. Here, we focus on a specific form of avian parental care, namely incubation of eggs. In some species parents can switch flexibly between breeding attempts from biparental to uniparental care or vice versa^14^. In others such flexibility seems less likely, for example because one sex (often the male) lacks a brood patch and hence cannot incubate effectively^21^. Flexibility may also be limited in species where both sexes possess a brood patch and share incubation roughly equally, because a single parent may not be able to attend the nest enough for embryos to develop until hatching, either because embryos cannot withstand fluctuating temperatures^19,22^, or because clutches that are left alone have a high probability of being depredated^23^. On the other hand, flexibility might be favoured by selection, because it would allow a single individual to obtain at least some reproductive success when its partner disappears (e.g. because of predation or disease).

Here, we used continuous incubation monitoring to investigate the occurrence of uniparental incubation in a sample of 15 shorebird species (Table 1), all of which are considered ‘obligate’ biparental incubators^24–26^. First, we report the frequency of uniparental incubation and describe how daily nest attentiveness (incubation constancy) changed from a biparental to a uniparental rhythm. Second, we compare the uniparental incubation rhythms of the biparental species (where both parents typically incubate) with the incubation rhythms of obligatory uniparental shorebird species with female-only incubation (pectoral sandpiper, *Calidris melanotos*) and with male-only incubation (red-necked phalarope, *Phalaropus lobatus*). Finally, we describe how many of the uniparentally incubated clutches succeeded (i.e. at least one chick hatched) and investigate whether hatching success was related to the start of the uniparental phase within the incubation period, to the duration of the uniparental phase, and to the median daily nest attendance during the uniparental phase.

**Table 1:** Overview of cases of uniparental incubation in nests of biparentally incubating shorebirds*.

Species	Scientific name	Population**	Total	Uniparental		♂only		Known outcome	Successful***	
			*N*	*N*	%	*N*	%	*N*	*N*	%
Western sandpiper	*Calidris mauri*	Barrow, Alaska	21	10	48%	8	80%	7	3	43%
Baird’s sandpiper	*Calidris bairdii*	Barrow, Alaska	18	8	44%	4	50%	6	5	83%
Long-billed dowitcher	*Limnodromus scolopaceus*	Barrow, Alaska	13	4	31%	4	100%	3	0	0%
American golden plover	*Pluvialis dominica*	Barrow, Alaska	23	6	26%	4	67%	4	0	0%
Semipalmated sandpiper	*Calidris pusilla*	Barrow, Alaska	183	36	20%	31	86%	31	4	13%
Black-tailed godwit	*Limosa limosa*	Selfoss, Iceland	5	1	11%	1	100%	1	1	100%
Black-tailed godwit	*Limosa limosa*	The Netherlands	4	—						
Redshank	*Tringa totanus*	The Netherlands	42	2	5%	2	100%	2	2	100%
Redshank	*Tringa totanus*	Selfoss, Iceland	2	—						
Dunlin	*Calidris alpina*	Barrow, Alaska	23	1	4%	1	100%	1	0	0%
Dunlin	*Calidris alpina*	Selfoss, Iceland	12	—						
Dunlin	*Calidris alpina*	Greenland	2	—						
Little ringed plover	*Charadrius dubius*	Czech Republic	17	—						
Semipalmated plover	*Charadrius semipalmatus*	Barrow, Alaska	8	—						
Common ringed plover	*Charadrius hiaticula*	Selfoss, Iceland	7	—						
Eurasian oystercatcher	*Haematopus ostralegus*	Selfoss, Iceland	7	—						
Whimbrel	*Numenius phaeopus*	Selfoss, Iceland	6	—						
Eurasian golden plover	*Pluvialis apricaria*	Selfoss, Iceland	4	—						
Ruddy turnstone	*Arenaria interpres*	Barrow, Alaska	1	—						

*Ordered according to % of nests with cases of uniparental incubation, and – within species – by total number of nests.

**For information on the study sites see ref.^29,36^.

***At least one egg hatched in successful nests.

## Results

### Occurrence of uniparental incubation

**Figure 1:**
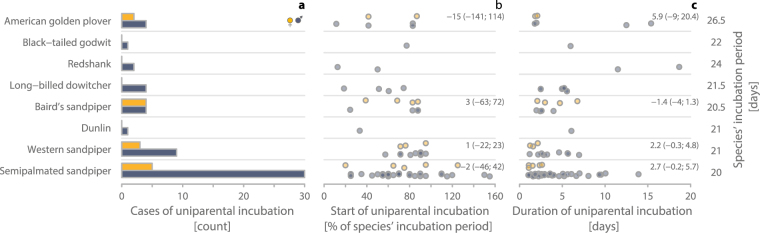
Uniparental incubation in eight biparental shorebirds according to sex. (**a**), Frequency of uniparental incubation by females and males (*N* = 70 cases from 68 nests). The species are ordered by phylogeny. (**b**) Distribution of the start of uniparental incubation within the incubation period, defined as the % of the species’ typical incubation period that had already passed (*N* = 69 cases from 68 nests). Values larger than 100% indicate uniparental incubation that started after the typical species-specific incubation period had passed without hatching (see Methods). (**c**) Distribution of the duration of uniparental incubation (*N* = 69 cases from 68 nests). Numbers on the right indicate the duration of the typical incubation period of the species in days (derived from ref.^24,25^). (**a**–**c**) Female uniparental incubation (; yellow), male uniparental incubation (; blue-grey). Data points are jittered to increase visibility. (**b**,**c**) For species with cases of both male and female uniparental incubation, we give the posterior estimates (medians) of the effect sizes and the 95% credible intervals from a posterior distribution of 5,000 simulated values generated by the ‘sim’ function in R^41^ (based on a separate linear model for each species with sex as predictor variable).

We found at least one case of uniparental incubation in 8 out of 15 biparental shorebird species (Table 1). Across species, the proportion of nests with uniparental incubation ranged from 4% to 48% (Table 1; median weighted by the total number of nests for a given species = 19%). Females incubated uniparentally less often than males (in 14 out of 70 cases, and in 4 out of 8 species; Fig. 1a and Supplementary Table 1 in ref.^27^).

Uniparental incubation started at various times within the incubation period (median = 71% of incubation period, range: 11–155%, *N* = 69 cases with known start of uniparental incubation from 68 nests of 8 species; Fig. 1b). Note that in some nests where eggs did not hatch both parents incubated beyond the typical incubation period (hence values >100%) before one parent deserted. The median remained similar (70%) after we excluded cases of uniparental incubation that started after the eggs were supposed to hatch (range: 11–95%, *N* = 62 cases from 60 nests of 8 species). Overall, the start of uniparental incubation within the incubation period was independent of sex (males differed from females by −5.7%, CI: −31% to 20%, *N* = 69) and likely varied little across species (’species’, added to the model as random intercept, explained only 7% of the phenotypic variance). Estimates of sex differences for each of the four species where both sexes incubated uniparentally are given in Fig. 1b.

Uniparental incubation lasted a median of 3 days (range: 1–19 days, *N* = 69 cases; Fig. 1c). Note that this is an underestimation, because in 10 nests we removed the monitoring system before incubation ended and in three nests only one parent incubated from the moment we found the nest. Overall, uniparental incubation by males lasted 2.4 days longer than uniparental incubation by females (CI: 0.5–4.3 days; *N* = 69 cases). However, species varied greatly in this respect (species explained 47% of the phenotypic variance). Estimates of sex differences for each species are given in Fig. 1c.

### Nest attendance during biparental and uniparental incubation

**Figure 2:**
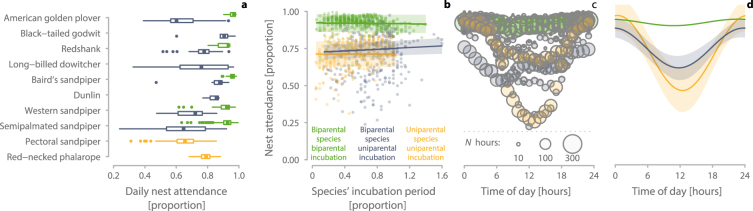
Daily nest attendance in biparental and uniparental shorebirds. (**a**) Distribution of biparental and uniparental daily nest attendance. Box plots depict median (vertical thick line inside the box), the 25^th^ and 75^th^ percentiles (box), the 25^th^ and 75^th^ percentiles ± 1.5 times the interquartile range or the minimum/maximum value, whichever is smaller (bars), and the outliers (dots). (**b**) Change in daily nest attendance across the incubation period (expressed as the proportion of the species’ typical incubation period). Each dot represents the nest attendance during one day. (**c**,**d**) Change in hourly nest attendance across the day. Circles (**c**) represent mean hourly observations for each species (circle size reflects sample size). (**b,d**) Lines with shaded areas indicate model predictions with 95% confidence intervals (Supplementary Table 2 & 4 in ref.^27^) based on the joint posterior distribution of 5,000 simulated values generated by the ‘sim’ function in R^41^. (**a**–**d**) Only nests that contain a uniparental incubation phase are included. Green () indicates biparental species during a biparental phase, blue-grey () biparental species during a uniparental phase, and yellow () uniparental species. *N*_a-b_ = 895 days and *N*_c-d_ = 23,258 hours from 87 nests of 10 species (65 nests of 8 biparental species, 22 nests of 2 uniparental species).

**Figure 3:**
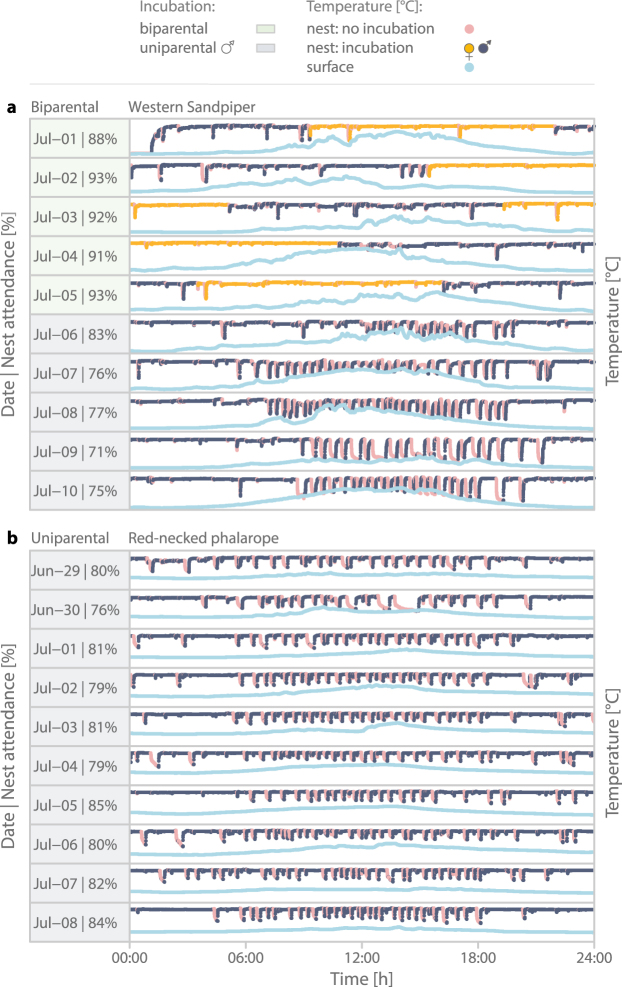
Example of a uniparental incubation rhythm by a biparental and a uniparental shorebird. (**a**) Biparental shorebird (western sandpiper) with a switch from biparental incubation (days marked green, ) to uniparental male-only incubation (grey, ). (**b**) Uniparental species (red-necked phalarope) with male-only incubation. (**a**,**b**) Pink () indicates nest temperatures, considered as no incubation; yellow () indicates nest temperatures considered as incubation while the female was on the nest and dark-blue () indicates when the male was on the nest (see Methods for details). Light-blue () indicates surface temperature in the vicinity of the nest. Temperatures were recorded every 5 s. Daily nest attendance is defined as the percentage of incubation readings (yellow + dark-blue;  + ) from all nest temperature readings for that day (pink + yellow + dark-blue;  +  + ).

After the switch from biparental to uniparental incubation, daily nest attendance decreased and was overall similar to the daily nest attendance observed in uniparental species (Figs 2a,b & 3). Daily nest attendance was similar across the incubation period in uniparental species and during biparental incubation (Fig. 2b), but it tended to increase over the incubation period during uniparental incubation in biparental species (Fig. 2b and Supplementary Table 2 in ref.^27^). However, individuals varied greatly in this respect (individual identity explained 35% of the variance, Supplementary Table 2 in ref.^27^). Also, nest attendance seemed to decrease over the incubation period in females of biparental species that incubated alone (Supplementary Fig. 1 and Supplementary Table 3 in ref.^27^).

The daily patterns of uniparental incubation in biparental species varied strongly between individuals. Some individuals continued to incubate as if their partner was still present, that is, they only incubated during ‘their’ bouts and left the nest unattended during the period when their partner would typically have incubated (e.g. actograms biparental_33, 38, 42, & 51 in Supplementary Actograms^27^). Other individuals developed an incubation rhythm similar to that of uniparental species, with continuous nest attendance during the colder parts of the day (the ‘night’) and intermittent incubation – presumably alternating with short feeding bouts – during the warmer part of the day (e.g. biparental_15, 70, 73, 76–7 in ref.^27^). However, within-individual variation in hourly nest attendance was far greater than the between-individual variation (within-individual [residual variance] = 53%, between-individual = 8% of variance; Supplementary Table 4). Indeed, some individuals first incubated as if their partner was still present and then switched to a ‘uniparental-like’ rhythm (e.g. biparental_26, 35, 42 in ref.^27^). When individuals continued to incubate as if their partner was still present, nest attendance was about 10–20% lower than when individuals incubated like uniparental species (Supplementary Actograms^27^). One male redshank *Tringa totanus* kept a ‘uniparental-like’ rhythm for 18 days (biparental_77 in ref.^27^), and one male semipalmated sandpiper *Calidris pusilla* for about 10 days (biparental_37 in ref.^27^).

In general, the 24-hour rhythm of uniparental incubation in biparental species closely resembled that of uniparental species with high nest attendance during the colder part of the day (‘night’) and lower nest attendance during the warmer part of the day (Figs 3 and 2c,d; for nest-specific patterns see Supplementary Fig. 2 in ref.^27^). In contrast, during biparental incubation, nest attendance was always high, with only a slight dip during the warmer part of the day (Fig. 2c,d). The rhythm of uniparental nest attendance in biparental species was similar for females and males (Supplementary Fig. 1 and Supplementary Table 5 in ref.^27^).

### Nest success for biparental species under uniparental incubation

**Figure 4:**
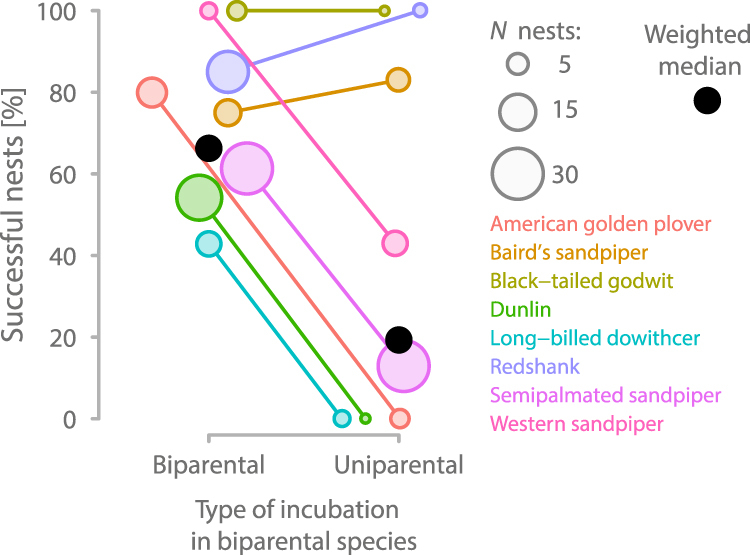
The proportion of successful nests of biparental species with biparental and uniparental incubation. Circles of different colours connected by lines represent different species. Black dots indicate median values, weighted by sample size (reflected by the size of the coloured circles). The data for biparental incubation come from the same populations as those for uniparental incubation (this study) and were extracted from ref.^29,36^.

Out of 55 uniparentally incubated nests (from 8 species) for which we knew the outcome, at least one chick hatched in 15 nests (27%; 5 species; Table 1). Four nests (7%) were depredated and in the remaining 36 nests (65%) the single parent also deserted before one of the eggs hatched. Nest success was independent of the cause or type of uniparental incubation (after we caught a parent, after a parent removal experiment^22^, temporal uniparental incubation period followed by another biparental period, unknown reason for uniparental incubation; Supplementary Table 6 in ref.^27^). The percentage of uniparentally incubated nests that were successful differed among species (ranging from 0–100%; Table 1). In 5 out of 8 species the percentage of successful uniparental nests was substantially lower than the percentage of successful nests that were incubated biparentally (Fig. 4), but for most species the sample size for uniparental incubation is small (Table 1).

**Figure 5:**
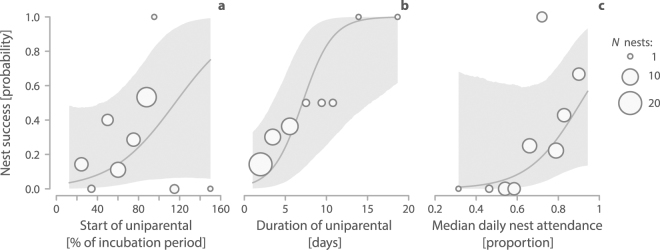
Predictors of nest success for biparental species under uniparental incubation. **(a**–**c**) Probability of nest success (hatching of at least one egg) as a function of (**a**) the start of uniparental incubation within the incubation period (expressed as the % of the species’ incubation period that had passed when uniparental incubation started, (**b**) the duration of uniparental incubation, and (**c**) the median daily nest attendance during uniparental incubation. Circles represent means for intervals spread evenly across the range of x-values; circle size reflects sample size. The solid lines depict the model-predicted relationships, the shaded area the 95% credible intervals based on the joint posterior distribution of 5,000 simulated values generated by the ‘sim’ function in R^41^; the predicted relationships stem from a binomial mixed-effect model (Supplementary Table 7 in ref.^27^), where the effect of the other predictors was kept constant. *N* = 50 nests with uniparental incubation from 8 biparental species.

Uniparentally incubated nests were more likely to be successful when the uniparental phase started later in the incubation period (Fig. 5a), when the uniparental phase lasted longer (Fig. 5b) and when median daily nest attendance during the uniparental phase was higher (Fig. 5c, Supplementary Table 7, see also Supplementary Fig. 3 in ref.^27^). Some parents successfully hatched their eggs when they continued uniparental incubation past the ‘normal’ incubation period (Supplementary Fig. 3 in ref.^27^), but individuals never succeeded when they started uniparental incubation after the ‘normal’ incubation period had already ended (Fig. 5a and Supplementary Fig. 3 in ref.^27^). The latter cases may arise if the probability of parental desertion increases when eggs fail to hatch around the expected date.

## Discussion

Our findings reveal that phases of uniparental incubation are not uncommon in biparental shorebirds, and challenge the belief that this necessarily leads to complete nest failure^25^. We found uniparental incubation in 8 out of 15 biparentally incubating shorebird species, and evidence of successful hatching of at least one egg in 27% of all uniparentally incubated nests with known outcome, from 5 out of 8 species with cases of uniparental incubation (Table 1). Reports of successful single-parent incubation from other species with ‘obligatory’ biparental incubation are rare^25^. Successful uniparental incubation in biparental incubators might truly be rare, but its frequency might be underestimated, because records of incubating parents throughout the entire incubation period are scarce^28,29^.

In biparental shorebirds, females typically desert their brood after hatching^25^. Here, we describe 68 cases (17% of 398 nests) where one parent disappeared prior to hatching, and indeed it was more often the female (80% of uniparental nests). In most of these nests, desertion is likely, but for two nests our video recordings revealed that one of the parents had been taken by a predator. We cannot exclude that this also occurred in other nests. Furthermore, when uniparental incubation occurred closer to hatching, we cannot exclude the possibility that the ‘deserting’ parent left to replenish its energy stores and later re-joined its partner to brood and guide the chicks. We have no evidence that this happens, but given that in three cases one parent deserted only for a few days during the incubation period and came back to incubate, it seems at least possible that this can also occur around the time of hatching.

We found substantial variation between nests in the timing of desertion/disappearance of one of the parents and in the total duration of uniparental incubation (Fig. 1b,c, and Supplementary Fig. 3 in ref.^27^). In 10% of cases uniparental incubation only started after the typical incubation period of the species had ended and such nests always failed. In other cases, individuals incubated uniparentally for at least half and up to nearly the entire incubation period (Fig. 1c and Supplementary Fig. 3 in ref.^27^). However, uniparental incubation often started in the second half of the incubation period (Fig. 1b and Supplementary Fig. 3 in ref.^27^) with a median duration of about three days (Fig. 1c and Supplementary Fig. 3 in ref.^27^). This suggests that individuals either continued incubating for a few days before deserting the nest, perhaps once realizing that they were incubating alone (which is in line with experimental findings^22^), or incubated uniparentally for a few days until the eggs started hatching.

Importantly, we found that clutches from biparental species that were uniparentally incubated for at least part of the incubation period can successfully hatch (Fig. 4 and Supplementary Fig. 3 in ref.^27^). The probability of hatching was higher when uniparental incubation started later in the incubation period (but before the expected hatch date), when uniparental incubation lasted longer (i.e. the parent did not give up) and when nest attendance by the single parent was higher (Fig. 5 and Supplementary Fig. 3 in ref.^27^). This suggests that in these biparental species one of the parents might benefit from deserting the nest – at least under certain conditions (e.g. depending on weather, food availability, condition or quality of the partner) – leaving the remainder of parental care to the partner. Indeed, some single parents were able or willing to incubate with a rhythm that closely resembled that of uniparental species (Fig. 3). The costs to those single parents remain unclear, but may include lower body condition, delayed migration and reduced probability of survival. We also emphasize that our study is not experimental, i.e. clutches that ended up with a single parent may not be a random sample and hence might differ from biparentally incubated clutches (e.g. in parental quality).

If individuals from species or populations that are considered obligatory biparental can behave as a uniparental species - with continuous incubation during the colder night and intermittent incubation interspersed with short foraging bouts during the warmer part of the day - then the potential exists for a flexible switch from biparental to uniparental care. Such flexibility may then lead to facultative biparental care^14,18,30^ or even to reduced or no care in one of the sexes. In turn, this could lead to a more flexible mating system including social polyandry and social polygyny^16,31^. Our results reveal that male uniparental incubation was more common than female uniparental incubation (Fig. 1). Thus, all else being equal, the evolution of polyandry would be more likely than the evolution of polygyny. It is worth investigating (a) whether flexible switches from biparental to (full) uniparental care occur in response to changing conditions (e.g. in response to warmer climate or to changes in mate availability), and (b) which factors determine who cares (e.g. population sex-ratio, individual quality or condition).

## Methods

### Data collection

Between 2011 and 2015, we recorded incubation at 398 nests from 19 populations of 15 biparentally incubating shorebird species (Table 1) using a radio frequency identification (‘RFID’) reader with a thin antenna loop fitted into a nest cup and connected to a data logger. Every 5 s, the logger registered the presence of a parent banded with a plastic flag containing a passive-integrated transponder^28,29^. Simultaneously, we monitored nest temperature and surface temperature next to the nest^28,29^.

In 2008 and 2009, we recorded uniparental incubation of 13 female pectoral sandpipers in Barrow, Alaska (71.32°N, 156.65°W) using an automated tracking system based on radio-telemetry (as described in ref.^29,32^). In 2015, we recorded uniparental incubation of 9 male red-necked phalaropes from Chukotka (64.75°N, 177.67°E) using nest and surface temperature probes^28,29^.

If birds were monitored already prior to or during laying (e.g. because an individual was equipped with a transmitter prior to incubation or because a bird was fitted with a passive transponder during a previous breeding attempt and its nest was found during laying), we estimated the start of incubation from the visualized raw data (‘actograms’, see Supplementary Actograms in ref.^29^), which show periods of continuous activity (prior to laying) or sporadic visits to the nest (e.g. during laying) that markedly contrast with subsequent incubation. If a nest was found during laying, we estimated the start of incubation by assuming that females laid one egg per day and started incubation when the clutch was completed (usually four, rarely three eggs). If nests were found with a full clutch, we estimated the start of incubation by subtracting the average incubation period of the species (derived from the literature, see Metadata in ref.^27^) from the hatch date; if the hatch date was unknown, we estimated the start of incubation based on the median height and angle at which the eggs of a given clutch floated in water, as described in detail elsewhere^33^. For one nest of American golden plover we lacked all relevant information; thus, we estimated the start of incubation as the median start of incubation of American golden plovers in the given population and year.

About half of the studied semipalmated sandpiper nests and 80% of the western sandpiper nests were protected against avian predators, at least for some days, using one of two enclosure types, both made of mesh wire (see Supplementary 1, Picture S1 in ref.^34^ and Supplementary Fig. 1 in ref.^22^). Although birds attending nests with an enclosure seemed to behave normally, we cannot exclude that the use of these enclosures influenced parental behaviour and the probability that a clutch hatched (independent of predation).

All field procedures were performed in accordance with the relevant guidelines and regulations, and approved by the local authorities.

### Extraction of incubation behaviour

We used local time for all incubation records calculated as UTC time + longitude of the nest × 24/360. For nests with temperature recordings, constant nest temperatures above the surrounding surface temperature were interpreted as continuous incubation; the start of incubation was determined from the steep increase in nest temperature, the end of incubation from a steep decrease in temperature (for detail see ref.^28^ and Scripts in ref.^27,35^).

For pectoral sandpiper nests with automated tracking, we used changes in the recorded signal strength from the radio-tag attached to the rump of the female: incubation was inferred whenever signal strength remained nearly constant (for details see ref.^32^ and Scripts in ref.^27^).

### Definition of uniparental incubation

A parent can either disappear/desert during its own incubation bout or when its partner is incubating (during the ‘off-nest’ bout). When a parent disappears while off-nest, its partner will typically be unaware of this and incubate the ‘regular’ incubation bout, here defined as the median incubation bout length observed in that population (see data in ref.^27^, derived from ref.^29,36^). When a parent disappears while incubating, its partner will typically come to the nest at the ‘expected’ change-over time and incubate its ‘regular’ incubation bout^22^. Then, the ‘deserted’ parent will often compensate for the absence of its partner, incubating during the period when its partner would typically have been on the nest, but then it may give up and desert the nest^22^. In this study, cases in which the ‘deserted’ parent only stays somewhat longer on the nest than usual are not included as cases of uniparental incubation.

Here, we define uniparental incubation as those cases where a single parent incubated for at least twice the median incubation bout of the population, excluding the parent’s first regular incubation bout. We also excluded the 6-hour period before the start of hatching or the 24-hour period before the chicks were found in the nest (hatched) or had left the nest. In this way we limited the data to true uniparental incubation periods, excluding (a) one prolonged incubation bout due to the partner’s absence, (b) cases where this bout was followed by complete nest desertion, and (c) periods that were confounded by hatching. Furthermore, including only longer periods of uniparental incubation allowed us to investigate the change in nest attendance within a day or over several days, i.e. from the period when the ‘deserted’ parent may still have been unaware of the partner’s absence or when it attempted to compensate for a possible delayed return of the partner^22^ to the period when the individual responds to the longer absence of the partner.

In total, we identified 70 periods of uniparental incubation from 68 nests after a parent naturally disappeared (either deserted the nest or died, which was usually unknown; *N* = 54 cases), after a parent deserted following capture and release (*N* = 13 cases), or after we experimentally removed a parent (*N* = 3 cases from semipalmated sandpiper^22^). Two nests had two uniparental incubation periods, because one of the parents was absent for several days, came back to incubate, but then permanently ‘deserted’. One nest had only one such ‘temporal’ uniparental incubation period.

### Definition of nest attendance

To compare incubation patterns between biparental and uniparental periods, and to compare uniparental incubation patterns between biparental and uniparental species, we used hourly and daily ‘nest attendance’ (also referred to as ‘incubation constancy’), defined as the proportion of time a bird actually incubated. We only included periods (either a particular hour or a particular day) when at least 75% of the total time was either biparental or uniparental incubation. For example, if a nest was biparentally incubated for 80% of a particular day, and then uniparentally for the remaining 20% of that day, we only included the 80% biparental data in our estimate of biparental daily nest-attendance. For estimates of nest attendance, we also excluded one complete nest and part of the data from two nests, because the temperature readings failed due to a dislocated probe. We further excluded two nests where the uniparental bird incubated only a single egg. Thus, our data set on uniparental incubation included 895 data points for daily nest attendance and 23,258 data points for hourly nest attendance from a total of 87 nests from 10 species (65 nests of 8 biparental species, 22 nests of 2 uniparental species).

### Statistical analyses

#### Nest attendance

We tested the difference in nest attendance between biparental and uniparental incubation using two linear mixed-effect models. The first model contained daily nest attendance as the response variable, and an interaction between two predictor variables: (1) day in the incubation period, defined as the proportion of the species’ typical incubation period (available in ref.^27^, derived from ref.^24,25^) that had already passed, and (2) incubation type (biparental incubation, uniparental incubation in biparental species, uniparental incubation in uniparental species). To control for non-independence of data points within species and nests, and during biparental or uniparental incubation, we included nest and species in interaction with incubation type (here with only two levels: biparental or uniparental) as random intercepts. To control for species- and nest-specific responses to day in the incubation period and to avoid an overconfident estimate of the effect of day in the incubation period^37^, we included day in the incubation period as a random slope.

The second model contained hourly nest attendance as the response variable, and time of day in interaction with incubation type (three levels as above) as predictors. To linearize the circular variable ‘time’ we first transformed time of day to radians and then fitted a sine and cosine function to those. Similar to the previous model, we included nest and species interaction with incubation type (biparental or uniparental) as random intercepts, and time of day as a random slope.

For those four biparental species, where we observed both female and male uniparental incubation, we used two additional models to test whether uniparental incubation patterns differed between the sexes. The first model contained daily uniparental nest attendance as the response variable, and day in the incubation period (defined as above) in interaction with sex as predictors. Nest and species were included as random intercepts and day in the incubation period as a random slope. The second model contained hourly uniparental nest attendance as the response variable, time of day (transformed to radians and represented by the sine and cosine) in interaction with sex as predictors, nest and species as random intercepts, and time of day as a random slope.

#### Nest success

For 55 biparental nests with phases of uniparental incubation we had information about the fate of the nest and for 51 of those also information about nest attendance. We thus used a binary mixed effect model to test whether nest success (binary response variable indicating whether at least one egg hatched or not) was related to (1) the start of the uniparental incubation phase within the incubation period (defined as above), (2) the duration of the uniparental incubation (in days), and (3) the median daily nest attendance during the uniparental phase. Species was included as a random intercept. The correlations between the three predictors were low (all |*r*_Pearson or Spearmen_| < 0.32, *N* = 50 nests for which data on all three predictors were available).

#### General procedure

We used R version 3.3.0 ^38^ for all statistical analyses and the ‘lme4’ package^39^ for fitting the mixed-effect models. We used the ‘sim’ function from the ‘arm’ package and a non-informative prior-distribution^40,41^ to create a sample of 5,000 simulated values for each model parameter (i.e. posterior distribution). We report effect sizes and model predictions by the medians, and the uncertainty of the estimates and predictions by the Bayesian 95% credible intervals represented by the 2.5 and 97.5 percentiles (95% CI) from the posterior distribution of 5,000 simulated or predicted values. We estimated the variance components using the ‘lmer’ or ‘glmer’ function from the ‘lme4’ package^39^ with maximum likelihood.

### Open data, codes and materials

All available at https://osf.io/3rsny ^27^.
